# Prediction Techniques on FPGA for Latency Reduction on Tactile Internet

**DOI:** 10.3390/s22093556

**Published:** 2022-05-07

**Authors:** Sérgio N. Silva, Lucileide M. D. da Silva, Leonardo A. Dias, Marcelo A. C. Fernandes

**Affiliations:** 1 Laboratory of Machine Learning and Intelligent Instrumentation, Federal University of Rio Grande do Norte, Natal 59078-970, Brazil; s.natansilva@gmail.com (S.N.S.); lucileide.dantas@escolar.ifrn.edu.br (L.M.D.d.S.); 2 Federal Institute of Education, Science and Technology of Rio Grande do Norte, Santa Cruz 59200-000, Brazil; 3 Centre for Cyber Security and Privacy, School of Computer Science, University of Birmingham, Birmingham B15 2TT, UK; l.a.dias@bham.ac.uk; 4 Department of Computer Engineering and Automation, Federal University of Rio Grande do Norte, Natal 59078-970, Brazil

**Keywords:** tactile internet, robotic, FPGA, latency

## Abstract

Tactile Internet (TI) is a new internet paradigm that enables sending touch interaction information and other stimuli, which will lead to new human-to-machine applications. However, TI applications require very low latency between devices, as the system’s latency can result from the communication channel, processing power of local devices, and the complexity of the data processing techniques, among others. Therefore, this work proposes using dedicated hardware-based reconfigurable computing to reduce the latency of prediction techniques applied to TI. Finally, we demonstrate that prediction techniques developed on field-programmable gate array (FPGA) can minimize the impacts caused by delays and loss of information. To validate our proposal, we present a comparison between software and hardware implementations and analyze synthesis results regarding hardware area occupation, throughput, and power consumption. Furthermore, comparisons with state-of-the-art works are presented, showing a significant reduction in power consumption of ≈1300× and reaching speedup rates of up to ≈52×.

## 1. Introduction

Tactile Internet (TI) enables the propagation of the touch sensation, video, audio, and text data through the Internet [1]. TI-based communication systems will provide solutions to more complex computational problems, such as human-to-machine interactions (H2M) in real time [2,3]. Therefore, TI is a new communication concept that allows transmitting skills through the Internet [4]. Several applications are available in the literature, such as virtual and augmented reality, industrial automation, games, and education [5].

Currently, the system’s latency is a major bottleneck for TI applications. Therefore, it is necessary to guarantee very low latency, as demonstrated in [5,6,7,8]. Studies indicate that TI applications’ latency varies from 1 to 10 ms in most cases or up to 40 ms in specific cases. Nevertheless, high latency can result in many problems, as stated in [7], such as cybersickness [9,10]. Several works have investigated methods to minimize the problems associated with the latency on TI applications, as presented in [1,11,12,13,14]. The work shown in [15] provides a comprehensive survey of techniques designed to deal with latency, which proposes prediction techniques as a solution to minimize the impacts caused by delays and loss of information. Thus, the system “hides the real network latency” by predicting the user’s behavior; notably, the proposal does not reduce the latency but predicts the system behavior, thus, enhancing the user experience’s quality.

Plenty of research areas, such as market, industry, stocks, health, and communication, have used forecasting techniques over the years [16,17,18,19,20,21,22]. However, these techniques are often implemented in software, increasing the latency in computer systems within tactile links due to the high computational complexity of the techniques and the large datasets to be processed.

Systems based on reconfigurable computing (RC), such as field-programmable gate arrays (FPGAs), have been proposed to overcome the processing speed limitations of complex prediction techniques [23]. In addition, FPGAs enable the deployment of dedicated hardware, enhancing the performance of computer systems within the tactile system. In addition, systems deployed with FPGAs proposed in the literature can reach 1000× speedup compared to software-based ones [24,25,26,27,28].

Therefore, we propose the parallel implementation of linear and nonlinear prediction techniques applied to the TI on reconfigurable hardware, that is, on FPGA. Hence, the main contributions of this work are the following:

- Parallel implementation of prediction techniques on FPGA without additional embedded processors.
- A detailed description of the modules implemented for the linear and nonlinear regression techniques on FPGA.
- A synthesis-based analysis of the system’s throughput, area occupation, and power consumption, using data from a robotic manipulator.
- An analysis of fixed-point precision against floating-point precision used by software implementations.

### Related Works

The use of RC for computationally complex algorithms is widely available in the literature. Prediction techniques based on machine learning (ML), such as multilayer perceptron (MLP), are proposed to assist the bandwidth allocation process on the server automatically [29,30,31]. However, the presented systems are local and may not be scalable for use in more complex networks with higher traffic due to the need for data from all communications to perform the techniques’ configuration and training steps. Therefore, linear prediction techniques have been proposed in [32,33] to avoid the loss of packages or errors.

Numerous works applied to TI are software-based implementations, such as cloud applications [34,35,36]. Usually, these software-based approaches are slower compared to hardware-based ones, thus affecting the data processing time of prediction techniques. As a result, some proposals were deployed on FPGA to increase the performance of manipulative tools [37,38,39,40], requiring accurate feedback [41,42,43,44].

Prediction techniques deployed on hardware, such as FPGAs, can reduce the latency in computer systems. In [45], an implementation of the quadratic prediction technique based on FPGA regression is proposed. In [46], a technique to detect epistasis based on logistic regression is implemented with an FPGA combined with GPU, achieving between 1000× to 1600× speedup compared to software implementations. In [47], an implementation of a probabilistic predictor on FPGA is proposed. Ref. [23] presented the hardware area occupation and processing time results for various RNA configurations of functions radial bases. Meanwhile, [48,49] demonstrate the feasibility of implementing algorithms based on deep learning (DL) using an RC-based platform.

Few studies explore linear regression applied to signal prediction on FPGAs or predictors applied in TI systems. However, there are proposals for machine learning (ML) techniques on FPGA. As an example, [50] proposes an MLP architecture for wheezing identification of the auscultation of lung sounds in real time. The MLP training step is performed offline, and its topology contains 2 inputs, 12 neurons in the hidden layer, and 2 neurons in the output layer (2–12–2). The architecture uses a 36-bits fixed-point implementation on an Artix-7 FPGA, achieving a sampling time of 8.63 ns and a throughput of 115.88 Msps.

The work presented in [51] uses an MLP on FPGA to perform the activity classification for a human activity recognition system (HAR) for smart military garments. The system has seven inputs, six neurons in the hidden layer, and five in the output layer (7–6–5). In addition, five versions of the architecture were implemented by varying the data precision. The analysis shows that the MLP designed with a 16-bit fixed-point is more efficient concerning classification accuracy, resource utilization, and energy consumption, reaching a sampling time of 270 ns using about 90% of the embedded multipliers and a throughput of 3.70 Msp.

Another MLP implemented on FPGA is proposed by [52] for real-time classification of gases with low latency. The MLP has 12 inputs, 3 neurons in the hidden layer, and 1 neuron in the output layer (12–3–1). In addition, the Levenberg–Marquardt backpropagation algorithm is used to perform offline training. The architecture was developed on Vivado using high-level synthesis (HLS) to optimize the development time and deployed on a Xilinx Zynq-7000 XC7Z010T-1CLG400 FPGA. Concerning the bit-width, a 24-bit signed fixed-point representation was used for the trained weight data with 20 bits on the fractional part. Meanwhile, 16-bit (14 bits on the fractional part) was used to deploy the output layer using the TanH function. A throughput of 539.7 ns was achieved.

In [53], an MLP was implemented for automatic blue whale classification. The MLP had 12 inputs, 7 neurons in the hidden layer, and 3 in the output layer (12–7–3). The backpropagation algorithm was used for an offline training process. The trained weight data were deployed using fixed-point representation with a 24-bit maximum length. The output function adopted was the logistic sigmoid function. The architecture was developed on a Xilinx Virtex 6 XC6VLX240T and Artix-7 XC7A100T FPGAs, reaching a throughput of 27.89 Msps and 25.24 Msps, respectively.

Unlike the literature works discussed, we propose linear and nonlinear prediction techniques designed on hardware for TI applications to reduce the latency. The linear techniques proposed are predictions based on linear regression using the floating-point standard IEEE 754. In addition, four solutions for different ranges of the regression buffer are presented. Regarding the nonlinear techniques, an MLP-BP prediction technique is proposed, using fixed-point representation, performed with online training. The Phantom Omni dataset is used to validate the implementations and compare them to software versions implemented on Matlab.

## 2. Proposal Description

**Figure 1:**
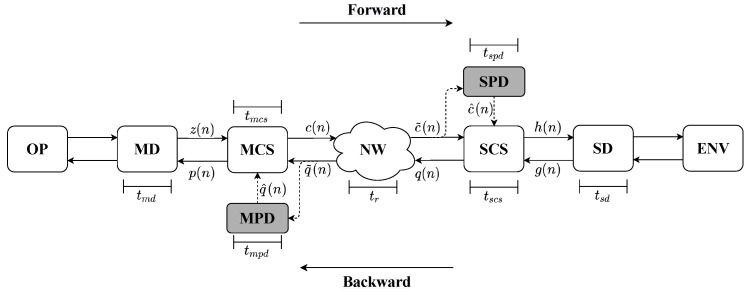
Block diagram illustrating the behavior of a generic Tactile Internet system that uses a parallel prediction method.

TI-based communication enables sending the sensation of touch through the Internet. The user, OP, interacts with a virtual environment or a physical tool, ENV, over the network. Figure 1 shows the general tactile internet system, with two devices interacting. The devices can be the most diverse, such as manipulators, virtual environments, and tactile or haptic gloves. The master device (MD) sends signals to the slave device (SD) during the forward flow. Meanwhile, the SD feedbacks the signals to the MD on the backward flow.

Each master and slave device has its subsystem, computational system, responsible for data processing, control, robotics, and prediction algorithms at each side of the communication process. MCS and SCS are the identifications for the master and the slave device computational systems, respectively. The total execution time of each of these blocks can be given by the sum of the individual time of each algorithm, assuming they are sequential.

The model adopted in this work considers that several algorithms constitute the computational systems, and each of them increases the system’s latency. Thus, the prediction process should be implemented in parallel to the other algorithms embedded in the MCS and SCS. This consideration aims to decouple prediction techniques from other algorithms, simplify the analysis, and to improve performance. Figure 1 presents a model that uses prediction methods in parallel with computational systems. The prediction modules, identified as MPD and SPD, have the same signal inputs as their respective computational systems, signals $\tilde{q}\left(n\right)$ and $\tilde{c}\left(n\right)$. In this project, the predictions performed use Cartesian values. The module MPD predicts a vector called $\hat{q}\left(n\right)$ upon receiving the input vector. This prediction has a processing time of $t_{mpd}$. Similarly, the SPD module predicts the $\hat{c}\left(n\right)$ vector on the slave side, with a prediction processing time of $t_{spd}$.

## 3. Prediction Methods

**Figure 2:**
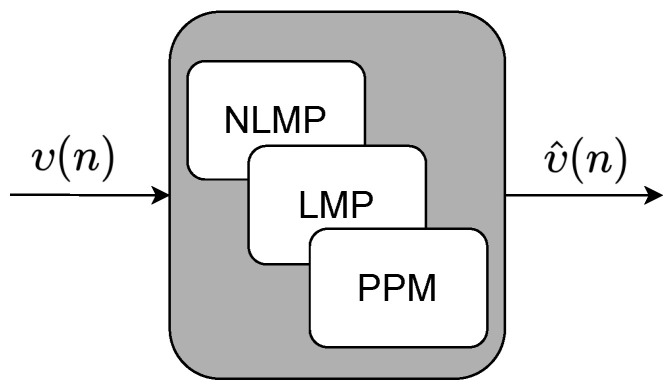
Structure of the prediction modules, MPD and SPD.

As shown in Figure 1, the modules responsible for the prediction system, called MPD and SPD, can be implemented in parallel with MCS and SCS computational systems. These prediction systems can execute nonlinear prediction methods (NLPM), linear prediction methods (LPM), or probabilistic prediction methods (PPM), as illustrated in Figure 2. We propose the implementation of linear regression and the multilayer perceptron with the backpropagation algorithm (MLP-BP).

As mentioned in the previous section, the system has two data streams, forward and backward, represented by the signal vectors c(n) and q(n). In this section, υ(n) represents the input samples, and $\hat{\upsilon }\left(n\right)$ represents the predicted samples for these two vectors in both streams.

Each prediction module can implement different prediction methods that can be applied for both Cartesian and joint coordinates, as described in [54]. The implementations can replicate the same technique multiple times. A replication index, NI, can be used as a metric to define the hardware capacity to implement multiple techniques in parallel. The NI value may vary according to the degree of freedom of the virtual environment or robotic manipulator model.

### 3.1. Linear Regression

The linear regression prediction model uses a set of M past samples to infer possible predicted data. It uses a set of observed pairs composed of the time marker, $t_{m}$, and the dependent variable, υ, that is, $\left(t_{m}\left(1\right),\upsilon \left(1\right)\right),\left(t_{m}\left(2\right),\upsilon_{m}\left(2\right)\right),\ldots ,(t_{m}\left(M-1\right),$υ(M−1)),$\left(t_{m}\left(M\right),\upsilon \left(M\right)\right)$. The regression can be defined by Equation (1),

(1) $$\hat{\upsilon }\left(n\right)={\hat{\beta }}_{0}\left(n\right)+{\hat{\beta }}_{1}\left(n\right)t_{m}\left(n\right),$$

where $\hat{\upsilon }\left(n\right)$ is the predicted value of υ(n), ${\hat{\beta }}_{0}\left(n\right)$ is the linear estimation coefficient, and ${\hat{\beta }}_{1}\left(n\right)$ is the coefficient of angular estimation for the same estimated sample. The parameter estimation process uses the principle of least squares [55]. Equations (2) and (3) indicate the coefficients,

(2) $${\hat{\beta }}_{0}\left(n\right)=\bar{\upsilon }\left(n\right)-{\hat{\beta }}_{1}\left(n\right)\bar{t_{m}}\left(n\right),$$

(3) $${\hat{\beta }}_{1}\left(n\right)=\frac{\sum_{j=0}^{M}\left(t_{m}\left(n-j\right)-\bar{t_{m}}\left(n\right)\right)\left(\upsilon \left(n-j\right)-\bar{\upsilon }\left(n\right)\right)}{\sum_{j=0}^{M}{\left(t_{m}\left(n-j\right)-\bar{t_{m}}\left(n\right)\right)}^{2}},$$

where $\bar{\upsilon }\left(n\right)$ and $\bar{t_{m}}\left(n\right)$ are the average values of the sample variables υ and $t_{m}$.

### 3.2. Multilayer Perceptron Networks

Commonly, complex problems are solved with machine-learning-based solutions, such as artificial neural networks (ANN). The mathematical structure of the ANN is composed of processing units called artificial neurons. The neurons can operate in a parallel and distributed manner [56]. Hence, ANN solutions can exploit the high parallelism degree provided by FPGAs.

#### 3.2.1. Architecture

Several applications based on neural networks use the architecture of an MLP-BP due to the ability to deal with nonlinearly separable problems [57]. Equation (4) represents the prediction function using the MLP technique, which uses *B* past samples of υ to generate the $\hat{\upsilon }\left(n\right)$ value, as follows:

(4) $$\hat{\upsilon }\left(n\right)=f\left(\upsilon_{n-1},\upsilon_{n-2},\ldots ,\upsilon_{n-B}\right),$$

where $\upsilon_{n-1},\upsilon_{n-2},\ldots \upsilon_{n-B}$ are the input values of the MLP and $\hat{\upsilon }$ is the MLP predicted output.

**Figure 3:**
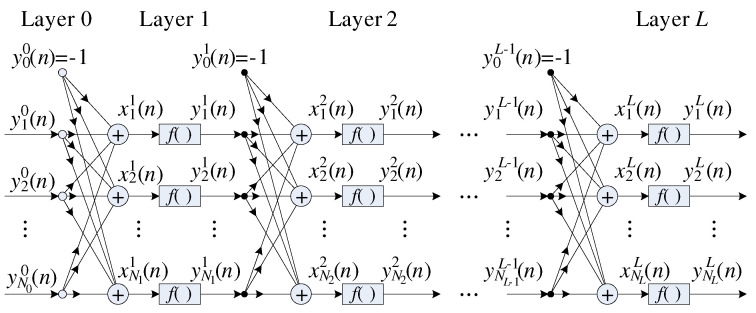
Structure of an MLP artificial neural network (ANN) with *L* layers.

**Figure 4:**
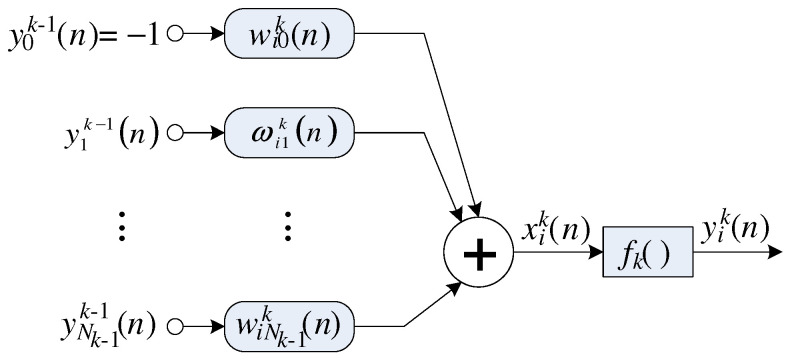
Structure of a neuron (perceptron) with $N_{k-1}+1$ inputs.

Equation (5) presents a generic MLP with *L* layers, where each *k*-th (k=1,…,L) layer can have $N_{k}$ neurons with $N_{k-1}+1$ inputs representing the number of neurons in the previous layer. The neurons from the *k*-th layer process their respective input and output signals through an activation function $f_{k}\left(•\right)$. At the *n*-th sample, this function is given by

(5) $$y_{i}^{k}\left(n\right)=f_{k}\left(x_{i}^{k}\left(n\right)\right),$$

where $y_{i}^{k}\left(n\right)\left(i=1,\ldots ,N_{k}\right)$ is the *i*-th neuron output in the *k*-th layer, and $x_{i}^{k}\left(n\right)$ can be defined as

(6) $$x_{i}^{k}\left(n\right)=\left(\sum_{j=1}^{N_{k}}w_{ij}^{k}\left(n\right)y_{j}^{k-1}\left(n\right)\right)-w_{i0}^{k}\left(n\right),$$

where $w_{ij}^{k}\left(n\right)$ is the synaptic weight associated with *j*-th input of the *i*-th neuron. Figure 3 illustrates the structure of an MLP ANN with *L* layers and Figure 4 illustrates the *i*-th neuron in the *k*-th layer.

The $f_{k}\left(•\right)$ function was defined by rectified linear unit (ReLU) function according to Equation (7):

(7) $$f_{k}\left(x\right)=max\left\{0,x\right\}.$$

The backpropagation algorithm is the training algorithm used with MLP.

#### 3.2.2. Backpropagation Training Algorithm

The weights are updated with the error gradient descent vector. At the *n*-th iteration, the *i*-th neuron error signal in the *k*-th layer is defined by

(8) $$e_{i}^{k}\left(n\right)=\left\{\begin{array}{cc} d_{i}\left(n\right)-y_{i}^{k}\left(n\right) & \;\mathrm{for}\;k=L\\ \sum_{j=0}^{N-1}w_{ij}^{k+1}\left(n\right)\delta_{i}^{k+1}\left(n\right) & \;\mathrm{for}\;k=1,\ldots ,L-1 \end{array}\right.,$$

where $d_{i}\left(n\right)$ is the desired value, and $\delta_{j}^{k+1}\left(n\right)$ is the local gradient for the *i*-th neuron in the (k+1)-th layer at the *n*-th iteration. Equation (9) describes the local gradient,

(9) $$\delta_{i}^{k+1}\left(n\right)=\left\{\begin{array}{cc} e_{i}^{k}\left(n\right)f^{\prime }\left(y\left(n\right)\right) & \;\mathrm{for}\;k=0,\ldots ,K-2 \end{array}\right.,$$

where $f^{\prime }\left(y\left(n\right)\right)$ is the derivative of the activation function.

The synaptic weights are updated according to the following:

(10) $$w_{ij}^{k}\left(n+1\right)=w_{ij}^{k}\left(n\right)+\eta \delta_{j}^{k}\left(n\right)y_{j}^{k}\left(n\right)+\alpha w_{ij}^{k}\left(n-1\right),$$

where η is the learning rate, α is the regularization or penalty term, and $w_{ij}^{k}\left(n+1\right)$ is the updated synaptic weight used in the next iteration.

## 4. Implementation Description

We propose an architecture using a 32-bit floating-point (IEEE754) format for the linear prediction technique. Throughout this section, we use the notation [F32]. For the MLP prediction technique proposed, we designed an architecture with a fixed-point format (varying the bit-width). We use the notation [sT.W] to represent the fixed-point values, where s represents the sign with 1 bit, T is the total number of bits, and W the number of bits in the fractional part. Therefore, the integer part of signed variables is T−W−1 bits long, while for unsigned variables it is T−W bits.

### 4.1. Linear Regression

The hardware architecture implemented for the linear prediction technique based on linear regression was based on Equations (1)–(3). All circuits in the structure use 32-bits floating-point precision.

**Figure 5:**
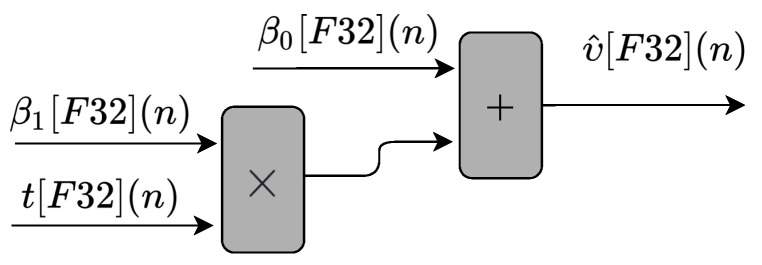
Block diagram representing the circuits implemented in hardware to perform the linear regression prediction technique, described in Equation (1).

The circuit shown in Figure 5 executes Equation (1). As can be observed, the circuit is composed of one multiplier and one adder. There are three input values, ($t_{m}\left[F32\right]\left(n\right)$, $\beta_{0}\left[F32\right]\left(n\right)$, and $\beta_{1}\left[F32\right]\left(n\right)$), and one output, ($\hat{\upsilon }\left[F32\right]\left(n\right)$).

**Figure 6:**
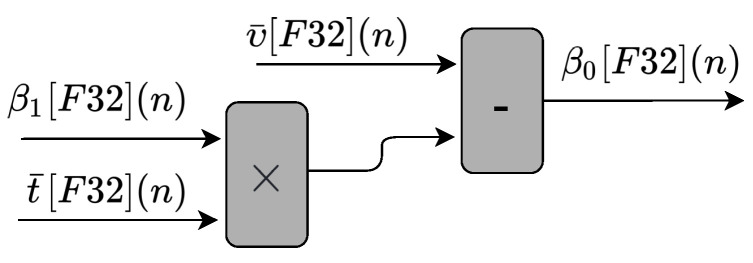
Block diagram representing the circuits implemented in hardware to obtain the $\beta_{0}$ variable used in the linear regression prediction technique.

To perform Equation (2), we use one multiplier and one subtractor, as shown in Figure 6. The circuit has three inputs values ($\bar{t_{m}}\left[F32\right]\left(n\right)$, $\beta_{1}\left[F32\right]\left(n\right)$, and $\bar{\upsilon }\left[F32\right]\left(n\right)$), and one output value ($\beta_{0}\left[F32\right]\left(n\right)$). The $\bar{t_{m}}\left[F32\right]\left(n\right)$ and $\bar{\upsilon }\left[F32\right]\left(n\right)$ inputs are the mean value of $t_{m}\left[F32\right]\left(n\right)$ and υ[F32](n), respectively.

**Figure 7:**
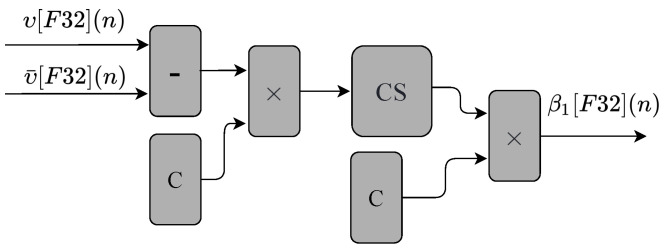
Block diagram representing the circuits and modules implemented in hardware to obtain the $\beta_{1}$ variable used in the linear regression prediction technique.

The circuit shown in Figure 7 performs Equation (3). As can be seen, the circuit is composed of two multipliers, one subtractor, one cascading sum module (CS), and two constant values (C). The constant values, C, were obtained empirically to simplify the existing division process in Equation (3). The circuit has two inputs values (υ[F32](n), $\bar{\upsilon }\left[F32\right]\left(n\right)$), and one output value ($\beta_{1}\left[F32\right]\left(n\right)$).

**Figure 8:**
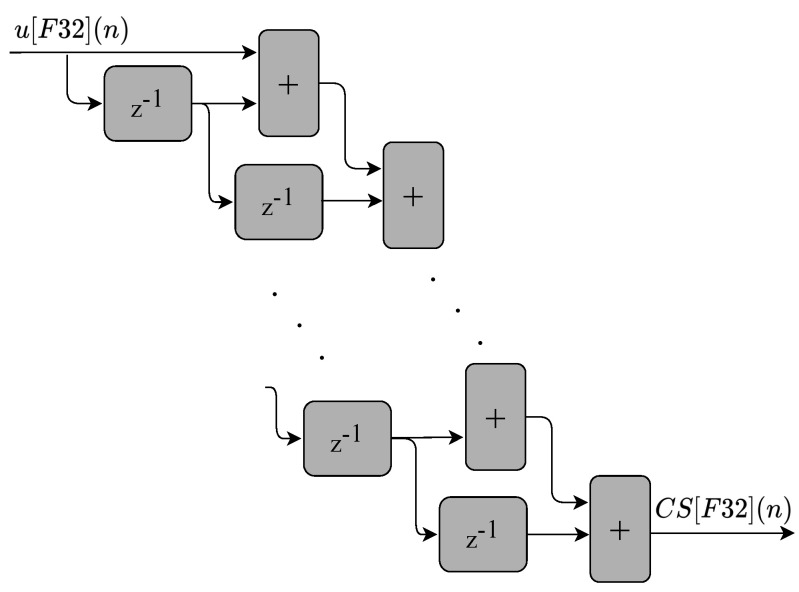
Block diagram representing the circuits used to implement the cascading sum (CS) hardware module. It receives u[F32](n) as input value (generated in the previous multiplier), and outputs the CS[F32](n) value.

**Figure 9:**
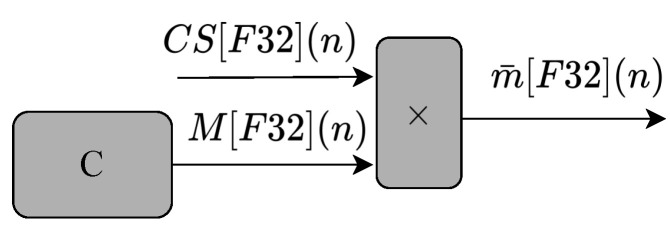
Block diagram representing the circuits for generating the mean values $\bar{t}\left[F32\right]\left(n\right)$ and $\bar{\upsilon }\left[F32\right]\left(n\right)$ used as inputs of the circuits shown in Figure 6 and Figure 7, respectively.

The cascading sum (CS) module shown in Figure 7 is implemented by the generic circuits shown in Figure 8. The cascading sum is also used as an input to calculate the mean values of t[F32](n) and υ[F32](n), as shown by the circuit illustrated in Figure 9.

### 4.2. Multilayer Perceptron

**Figure 10:**
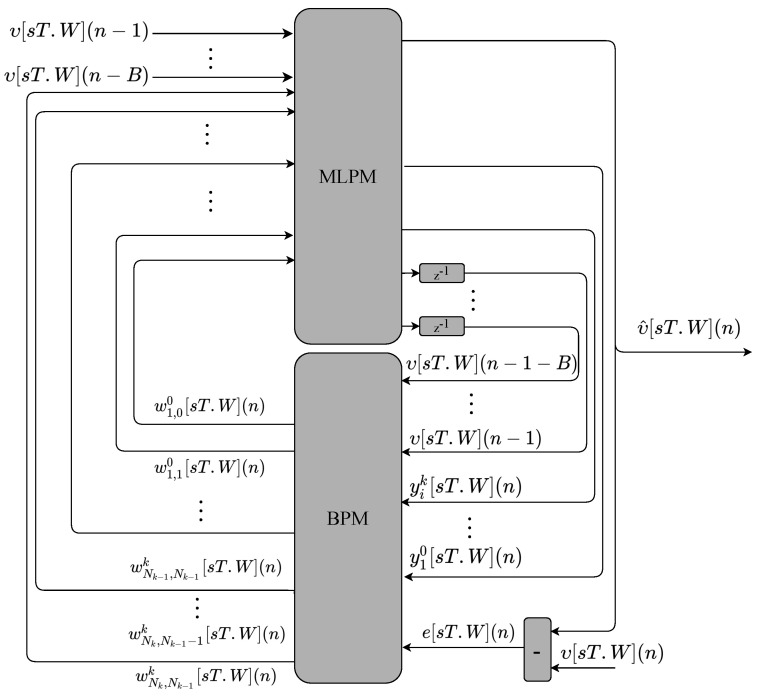
Main hardware modules implemented to perform the MLP-BP.

**Figure 11:**
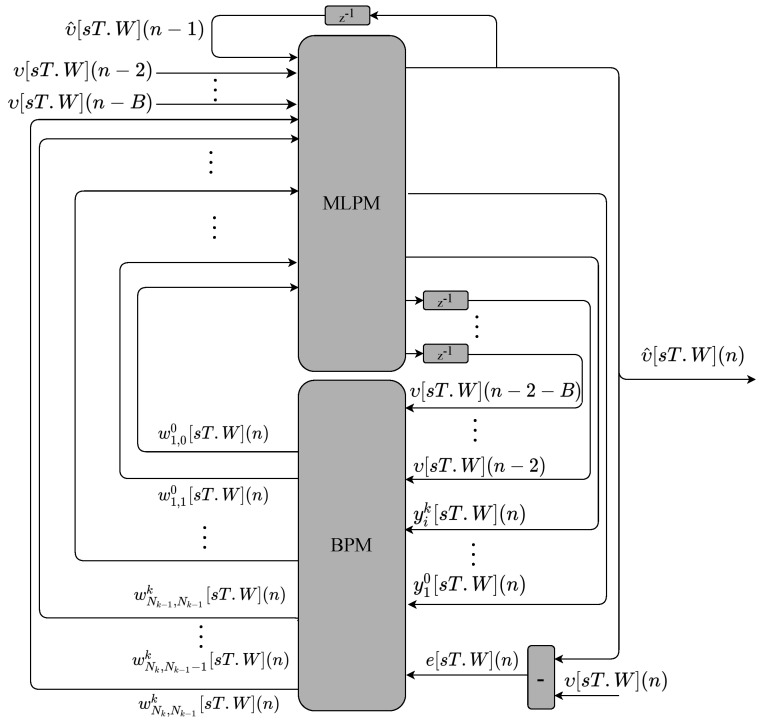
Main hardware modules implemented to perform the RMLP-BP.

The main modules that perform the multilayer perceptron with the backpropagation training (MLP-BP) and the multilayer perceptron with recurrent output (RMLP-BP) are shown in Figure 10 and Figure 11, respectively. The hardware structures are similar. The main difference between them is that the first input signal of the RMLP-BP is a feedback of the output signal. As can be observed, there are two main modules called multilayer perceptron module (MLPM) and backpropagation module (BPM). Both modules implement the variables in fixed-point format.

The MLPM module for the MLP-BP proposal (Figure 10) has *B* inputs from previous instants of the υ variable. The MLPM for the RMLP-BP proposal (Figure 11) has B−1 inputs from previous instants of the υ variable. The MLPM module forwards the υ inputs to the BPM modules with a unit delay. The BPM also receives the MLPM neurons output signal, $y_{i}^{k}\left[sT.W\right]\left(n\right)$, as well as the desired MLP output value characterized by an error signal, e[sT.W](n). Given that the desired value is equal to the current sample of a time series, that is $d_{i}\left[sT.W\right]\left(n\right)=\upsilon \left[sT.W\right]\left(n\right)$, the error can be defined as $e\left[sT.W\right]\left(n\right)=\upsilon \left[sT.W\right]\left(n\right)-\hat{\upsilon }\left[sT.W\right]\left(n\right)$. Finally, during the neuron update process, the BPM defines the new weight values, $w_{N_{k},N_{k-1}}^{k}\left[sT.W\right]\left(n\right)$, and forwards them back to the MLPM.

#### 4.2.1. Multilayer Perceptron Module (MLPM)

**Figure 12:**
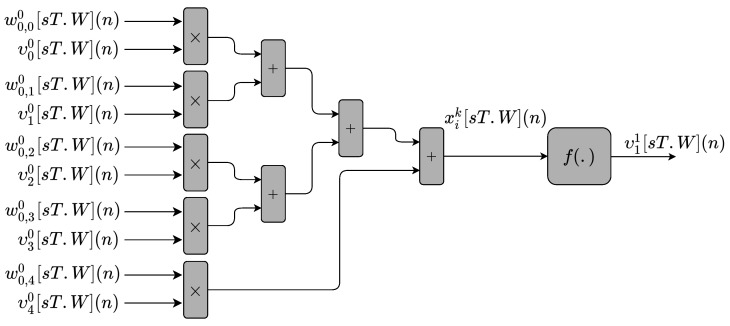
Block diagram representing the circuits used to implement the neurons of the MLPM module for both the MLP-BP and RMLP-BP.

Figure 12 presents the hardware implementation of the neurons of an ANN structure (Figure 3). MLPM module circuits implement the neurons based on Equations (5) and (6). As can be observed, it is a semi-parallel implementation for one neuron with ten inputs values, of which four are the υ[sT.W](n) and one bias ($\upsilon_{0}^{0}\left[sT.W\right]\left(n\right)$) values, while the remaining five inputs are the weight values, $w_{N_{k},N_{k-1}}^{k}\left[sT.W\right]\left(n\right)$. The sequential combination of adders and multipliers generates the output $x_{i}^{k}\left[sT.W\right]\left(n\right)$. The hidden layers of the network also use the structure described.

**Figure 13:**
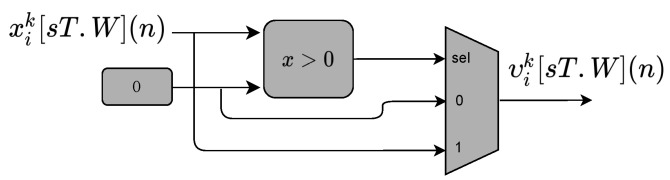
Block diagram representing the circuits used to implement the ReLU function (f(.)) submodule in Figure 12.

As mentioned in Section 3.2.1, the output layer uses the ReLU activation function. Figure 13 shows its hardware implementation. The signal $x_{i}^{k}\left[sT.W\right]\left(n\right)$ is the input of the nonlinear function described in Equation (7). The linear combination of weight and hidden layer output provides the neural network output.

#### 4.2.2. Backpropagation Module (BPM)

**Figure 14:**
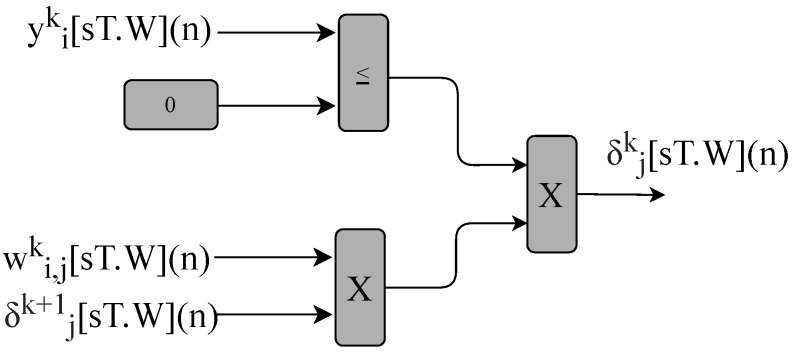
Block diagram representing the circuits used to obtain the hidden layers gradient implemented in the BPM module for the MLP-BP and RMLP-BP.

The BPM defines the error gradient and updates the neurons’ weights. The error gradient, e[sT.W](n), described in Equations (8) and (9), is performed by the circuits shown in Figure 14. The signals $w_{i,j}^{k}\left[sT.W\right]\left(n\right)$, $y_{i}^{k}\left[sT.W\right]\left(n\right)$ and $\delta_{j}^{k+1}\left[sT.W\right]\left(n\right)$ define the $\delta_{j}^{k}\left[sT.W\right]\left(n\right)$ gradient.

**Figure 15:**
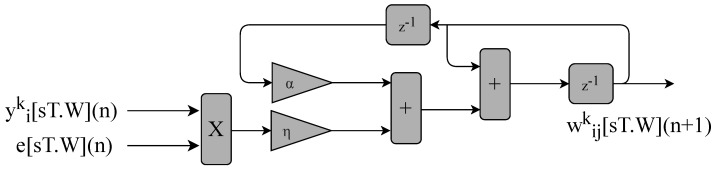
Block diagram representing the circuits used for updating the neurons’ weights implemented in the BPM module of the the MLP-BP and RMLP-BP.

The circuit shown in Figure 15 calculates the MLP neurons’ weights, as previously described in Equation (10). It consists of two inputs, $y_{i}^{k}\left[sT.W\right]\left(n\right)$ and e[sT.W](n), and two constants, α and η. The constants are defined using the fixed-point format [sT.W].

Table 1 summarizes the value used for each parameter in the MLP-BP and RMLP-BP hardware implementation. It is essential to mention that the training parameter was empirically defined.

## 5. Synthesis Results

This section presents synthesis results for linear and nonlinear prediction techniques. Three key metrics are analyzed: area occupation, throughput, and power consumption. This work’s throughput ($R_{s}$) has a 1:1 ratio with frequency (MHz). All synthesis results analyzed here use a Xilinx Virtex-6 xc6vlx240t-1ff1156 FPGA, with 301,440 registers, 150,720 6-bits look-up tables (LUTs), and 768 digital signal processors (DSPs) that can be used as multipliers.

Firstly, we carried out analyses for the linear regression technique varying the M value from 1 to 3, 6, and 9, implemented in a 32-bit floating-point format. Secondly, we present the synthesis analysis values for MLP-BP using signed fixed-point configurations with the following bit widths: 18.14, 16.12, and 14.10. Finally, we also provide an analysis by increasing the number of implementations (NI) in parallel from 1 to 3 and 6, thus, increasing the number of variables processed in parallel.

### 5.1. Linear Prediction Techniques

Table 2, Table 3 and Table 4 show the synthesis results for the linear regression prediction technique with 1, 3, and 6 parallel implementations, respectively. The first column of each table highlights the M value. The second to seventh columns present the area occupation on the FPGA. The second and third display the number of registers/flip-flops (NR) and their percentage (PNR), and the fourth and fifth, the number of LUTs (NLUT) and their percentage (PNLUT). Finally, the sixth and seventh indicate the number of multipliers (NMULT) and their percentage (PNMULT). The last two columns show the processing time, $t_{s}$, in nanoseconds (ns), and the throughput, $R_{s}$, in mega-samples per second (Msps).

**Figure 16:**
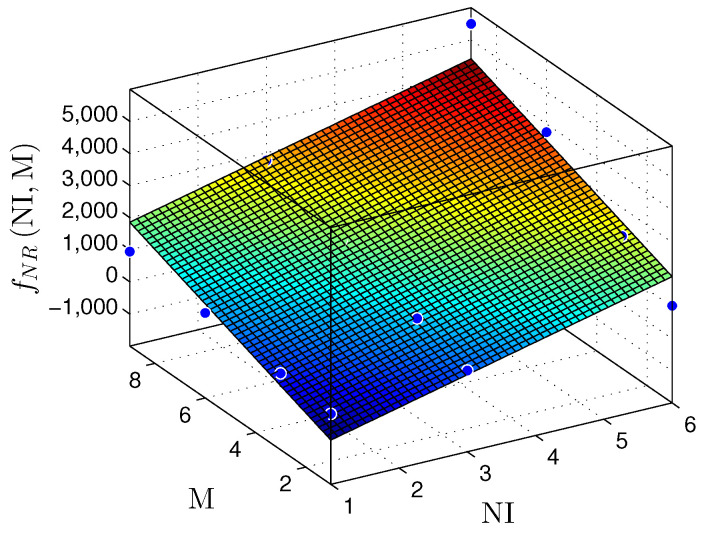
Plane $f_{NR}\left(NI,M\right)$ that estimates the NR, as a function of the NI and M, for the linear regression prediction technique.

**Figure 17:**
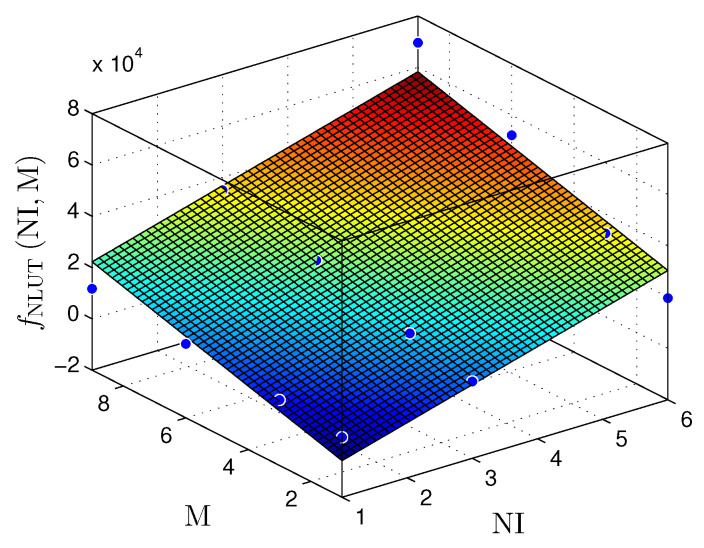
Plane $f_{NLUT}\left(NI,M\right)$ that estimates the NLUTs, as a function of the NI and M, for the linear regression prediction technique.

**Figure 18:**
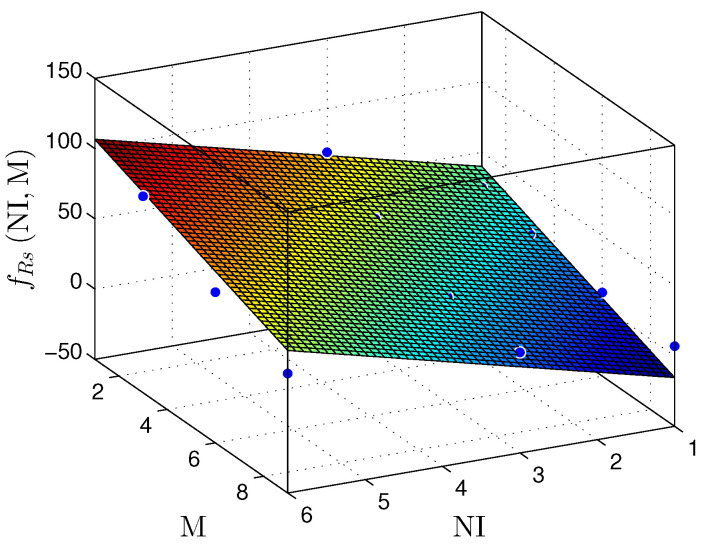
Plane $f_{R_{s}}\left(NI,M\right)$ that estimates the throughput, $R_{s}$, as a function of NI and M, for the linear regression prediction technique.

To demonstrate the linear behavior of our hardware proposal, we provide a linear regression model for Table 4. Figure 16, Figure 17 and Figure 18 show NR, NLUT, and $R_{S}$ results. It is essential to mention that linear regression models return a coefficient of determination called $R^{2}$. The $R^{2}$ rate represents the quality of the linear regression model, i.e., it demonstrates the obtained data variance. Commonly, $R^{2}$ is expressed on a scale from 0% to 100% (or a scale from 0 to 1 for normalized values). Concerning the NR, the plane $f_{\mathrm{NR}}\left(\mathrm{NI},M\right)$ can be described by

(11) $$f_{\mathrm{NR}}\left(\mathrm{NI},M\right)\approx -1439+510.7\times \mathrm{NI}+309.5\times M;$$

the coefficient of determination is $R^{2}=0.8553$.

Meanwhile, the NLUT, shown in the plane $f_{\mathrm{NLUT}}\left(\mathrm{NI},M\right)$, can be defined by

(12) $$f_{\mathrm{NLUT}}\left(\mathrm{NI},M\right)\approx -16,360+7210\times \mathrm{NI}+3487\times M;$$

and $R^{2}=0.8863$.

Finally, the the plane $f_{R_{s}}\left(\mathrm{NI},M\right)$ presents the throughput in Msps, is presented in the plane $f_{R_{s}}\left(\mathrm{NI},M\right)$, and is described as

(13) $$f_{R_{s}}\left(\mathrm{NI},M\right)\approx 33.4+13.35\times \mathrm{NI}-6.896\times M,$$

and $R^{2}=0.8372$.

According to the $t_{s}$ results presented in Table 2, Table 3 and Table 4 and Figure 18, a significant reduction in throughput is noticeable as M increases. Increasing the number of circuits in the cascading sum (CS) submodule results in a more significant critical path and, thus, a more considerable sampling time ($t_{s}$). However, the throughput increases proportionally to NI for a fixed value of M.

It is observable that there is a linear increase in the number of resources used as M and the NI grow. As presented in Table 4, for NI=6 and M=9, 46% of the NLUT are occupied. On the other hand, for smaller values such as M=3 and NI=6, the NLUT occupied is 21.53%. Additionally, it is possible to increase the NI using the remaining resources. However, there is no guarantee that there will not be large throughput losses.

Therefore, it is relevant to mention that the parallel FPGA implementations of the linear regression can achieve high throughput, as required in the TI scenario. On the other hand, these implementations result in high hardware area occupation. Considering that TI is still under development, high processing speed and intelligent use of resources are crucial.

### 5.2. Nonlinear Prediction Techniques

Commonly, MLP-based implementations use the hyperbolic tangent function. However, using this function resulted in a 28% occupation of the FPGA memory primitives for an MLP of four inputs, four neurons in the hidden layer, and one neuron in the output layer (with N=1). For N=6, it could occupy ≈68% of the memory primitives, making the *tanh* function unfeasible due to its high hardware implementation cost. The activation function that we use in this work is ReLU, since its hardware implementation does not require the use of memory primitives. As previously described, Equation (7) describes the ReLU function.

Table 5 and Table 6 show the hardware area occupation and throughput results for the MLP-BP and RMLP linear prediction techniques. The analyses for both techniques use a Virtex-6 FPGA. As presented in the first columns (T.W), they are implemented for different unsigned fixed-point bit widths.

**Figure 19:**
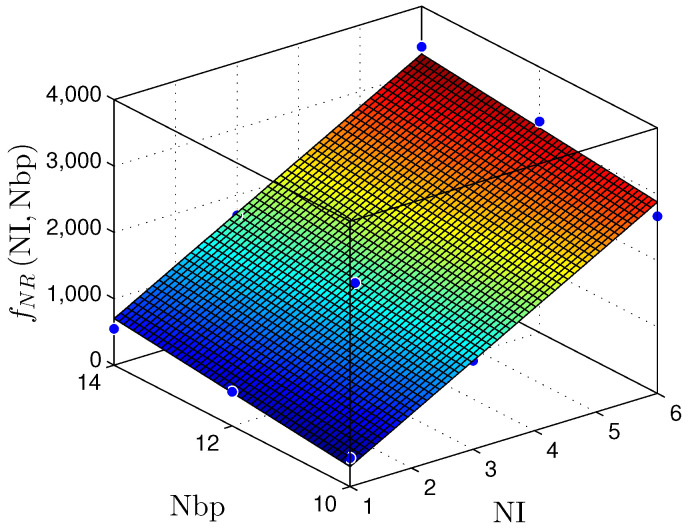
Plane, $f_{NR}\left(NI,W\right)$, found to estimate the number of registers, NR, as a function of the number of implementations, NI, and the number of bits in fractional part W for ML-based prediction techniques.

**Figure 20:**
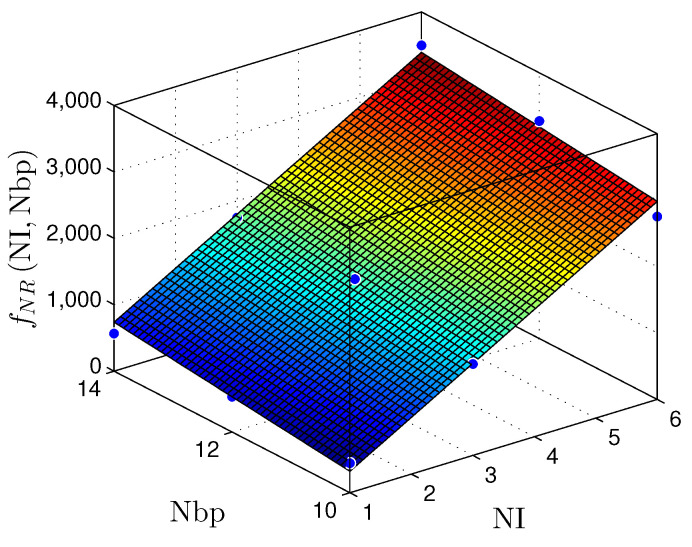
Plane, $f_{NR}\left(NI,W\right)$, found to estimate the number of registers, NR, as a function of the number of implementations, NI, and the number of bits in fractional part W for RLMP-based prediction techniques.

The results displayed in Table 5 and Table 6 make it possible to plot surfaces demonstrating the hardware behavior concerning the area occupation and throughput. Figure 19 and Figure 20 present the relationship between the NI and the number of bits in the fractional part (W) with the number of registers (NR) for the MLP and RMLP, respectively.

The $f_{\mathrm{NR}}\left(\mathrm{NI},W\right)$ planes can be expressed by

(14) $$f_{\mathrm{NR}}^{MLP}\left(\mathrm{NI},W\right)\approx -1439+510.7\times \mathrm{NI}+309.5\times W,$$

with $R^{2}=1$, and

(15) $$f_{\mathrm{NR}}^{RMLP}\left(\mathrm{NI},W\right)\approx -1237+531.4\times \mathrm{NI}+103\times W,$$

and $R^{2}=0.9835$.

**Figure 21:**
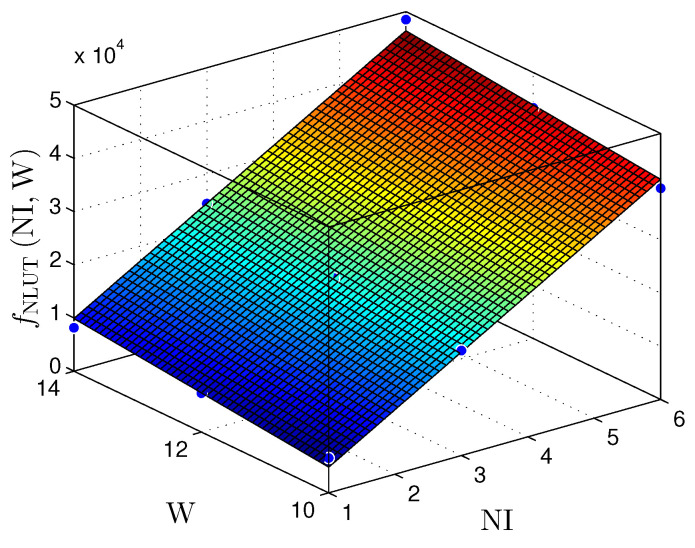
Plane, $f_{NLUT}\left(NI,W\right)$, found to estimate the number of LUTs, NLUT, as a function of the number of implementations, NI, and the number of bits in fractional part *W* for MLP-based prediction techniques.

**Figure 22:**
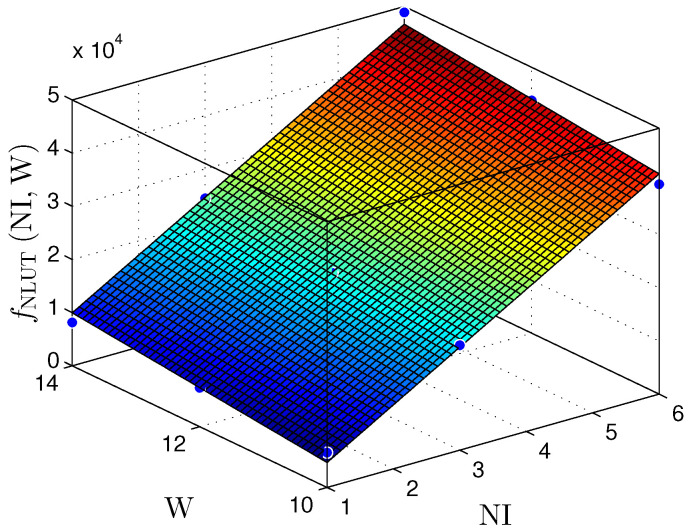
Plane, $f_{NLUT}\left(NI,W\right)$, found to estimate the number of LUTs, NLUT, as a function of the number of implementations, NI, and the number of bits in fractional part W for RMLP-based prediction techniques.

Figure 21 and Figure 22 present the relationship between the NI and the number of bits in the fractional part (W) with the number of LUTS (NLUTS) for the MLP and RMLP, respectively.

The $f_{\mathrm{NLUT}}\left(\mathrm{NI},W\right)$ planes can be expressed by

(16) $$f_{\mathrm{NLUT}}^{MLP}\left(\mathrm{NI},W\right)\approx -15,050+7305\times \mathrm{NI}+1260\times W,$$

for $R^{2}=0.9935$, and

(17) $$f_{\mathrm{NLUT}}^{RMLP}\left(\mathrm{NI},W\right)\approx -15,750+7342\times \mathrm{NI}+1312\times W,$$

for $R^{2}=0.9899$.

**Figure 23:**
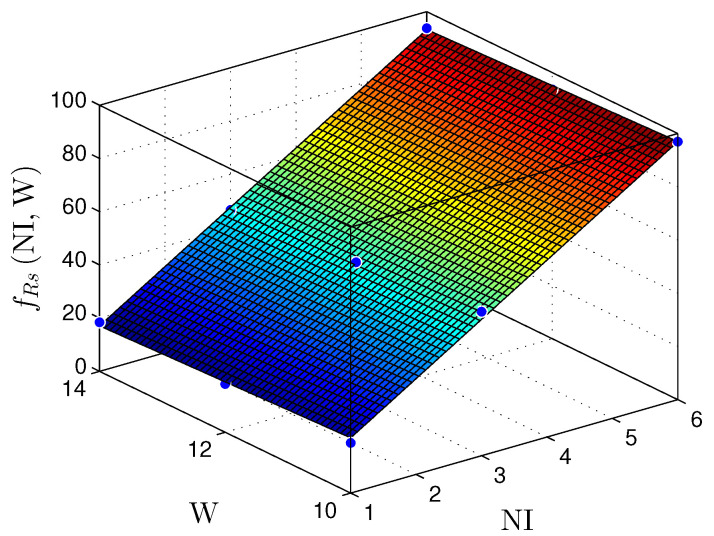
Plane, $f_{R_{s}}\left(NI,W\right)$, found to estimate the number of registers, $R_{s}$, as a function of the number of implementations, NI, and the number of bits in fractional part *W*, for MLP-based prediction techniques.

**Figure 24:**
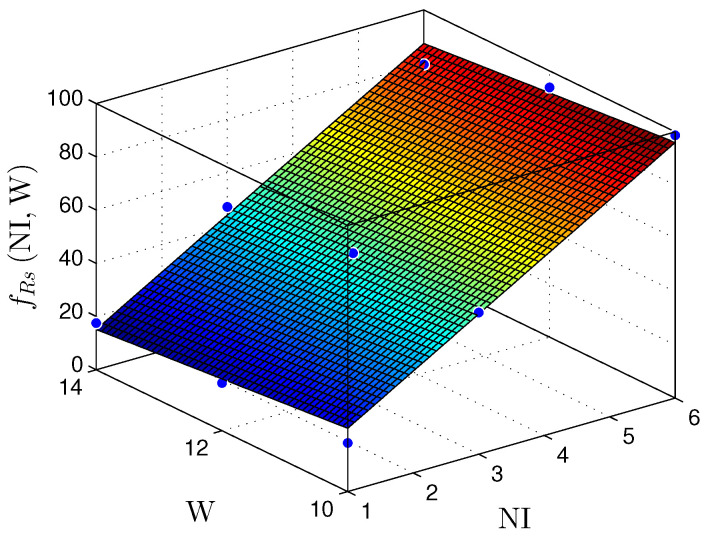
Plane, $f_{R_{s}}\left(NI,W\right)$, found to estimate the number of registers, $R_{s}$, as a function of the number of implementations, NI, and the number of bits in fractional part W, for RMLP-based prediction techniques.

Figure 23 and Figure 24 present the relationship between the NI and the number of bits in the fractional part (W) with the throughput ($R_{s}$) for the MLP and RMLP, respectively.

Equations (18) and (19) characterize the $f_{R_{s}}\left(\mathrm{NI},W\right)$ planes, for a throughput in Msps, as

(18) $$f_{R_{s}}^{MLP}\left(\mathrm{NI},W\right)\approx 14.92+15.24\times \mathrm{NI}-0.93\times W,$$

for $R^{2}=1$, and

(19) $$f_{R_{s}}^{RMLP}\left(\mathrm{NI},W\right)\approx 31.04+14.45\times \mathrm{NI}-2.17\times W,$$

for $R^{2}=1$.

Regarding the throughput ($R_{s}$) presented in Table 5 and Table 6, it is observable that the $R_{s}$ does not vary significantly for a fixed NI and a varying bit width (T.W). For a fixed bit width (T.W) and a varying NI, the throughput values have a linear increase proportional to the NI value. Nevertheless, it is also necessary to mention that the $t_{s}$ value has a low variance because the MLP and BP structures adapt well to parallelism. Hence, the circuit provides good scalability without considerable performance losses. Compared to the linear regression discussed in Section 5.1, the MLP shows better flexibility.

The area occupation decreases as the bit width (T.W) and NI parameters also decrease. Reducing these parameters also reduces the modules’ circuits to store or process data. The multipliers (NMULT) are the most used resource, reaching up to ≈42% of occupation when NI=6. In addition, the MLP and RMLP result in a similar hardware area occupation, using less than 43%, 27%, and 2% of multipliers, LUTs, and registers, respectively. Given that, or the current design and chosen FPGA, the maximum value of NI feasible to implement would be 9 or 10. The throughput would remain close to the current range. Nevertheless, this analysis used only the Virtex-6 DSPs. It is important to emphasize that the available LUTs can implement multipliers, permitting an increase in the parallelization degree and throughput.

We also performed the synthesis for the MLP and BP algorithms separately to verify the hardware impact of each of them. Table 7 presents an MLP-only implementation, while Table 8 presents a BP-only implementation. Given that most of the works in the literature do not implement the BP or any training algorithm on hardware, we provide a complete analysis of the modules implemented separately. The MLP, for NI =6, occupies only 3.82% and 19.53% of the LUTs and multiplies (PNMULT), respectively. It also achieved a throughput of ≈188 Msps. Hence, the low resource usage shows that our approach provides good scalability and high performance for applications that do not require online training and only use the MLP module.

The synthesis results show that the hardware proposal occupies a small hardware area. As can be seen, the MLP uses less than 20% and 4% of multipliers and LUTs, respectively. Meanwhile, the BP occupies less than 4% multipliers and LUTs and reaches more than 39 Msps. Thus, it is possible to increase the architecture parallelization degree due to the unused resources, consequently enabling the acceleration of several applications that relies on massive data processing [58]. In addition, the unused resources can also be used for robotic manipulators with more degrees of freedom and other tools [59]. The low hardware area occupation also shows that smaller, low-cost, and low-consumption FPGAs can fit our approach for IoT and M2M applications [60].

Therefore, for the linear and nonlinear regression with BP implementations, the throughput results reached values up to ≈98Msps. These values make it possible to use these solutions in problems with critical requirements, such as TI applications [9,10,29,30,31]. Figure 19, Figure 20, Figure 21, Figure 22, Figure 23 and Figure 24 show that the MLP and RMLP techniques have similar results for NR, NLUT, and $R_{s}$. The similarity observed between the results is expected due to the RMLP architecture being similar to the MLP, except for the input $\hat{\upsilon }\left[sT.W\right]\left(n\right)$, which is now delayed by a time sample $t_{s}$. Therefore, the following sections will only focus on the MLP and MLP-BP results, as it provides better scalability for increasing the NI.

## 6. Validation Results

**Figure 25:**
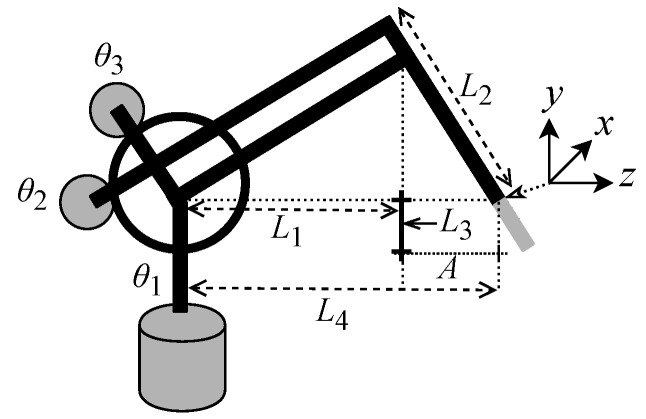
Structure of 3-DOF Phantom Omni robotic manipulator.

This work uses bit-precision simulation tests to validate the proposed hardware designs for the prediction techniques described in the previous section. Bit precision simulation is performed by a dynamic nonlinear system characterized by a robotic manipulator system with 6 degrees of freedom (DOF), i.e., rotational joints, called Phantom Omni [61,62,63,64]. Nonetheless, only the first three joints are active [64]. Therefore, the Phantom Omni can be modeled as a three-DOF robotic manipulator with two segments ($L_{1}$ and $L_{2}$) interconnected by three rotary joints ($\theta_{1}$, $\theta_{2}$, and $\theta_{3}$), as shown in Figure 25.

Based on the description provided by [63], the Phantom Omni parameters on the simulations carried out were defined as follows: $L_{1}=0.135\;\mathrm{mm}$; $L_{2}=L_{1}$; $L_{3}=0.025\;\mathrm{mm}$; and $L_{4}=L_{1}+A$ for A=0.035mm. In addition, the dynamics of the Phantom Omni can be described by nonlinear, second-order, and ordinary differential equations, as follows:

(20) $$M\left(\theta (t)\right)\ddot{\theta }\left(t\right)+C\left(\theta \left(t\right),\dot{\theta }\left(t\right)\right)\dot{\theta }\left(t\right)+g\left(\theta (t)\right)-f\left(\dot{\theta }\left(t\right)\right)=\tau \left(t\right),$$

where θ(t) is the vector of joints expressed as

(21) $$\theta \left(t\right)={\left[\begin{array}{ccc} \theta_{1}\left(t\right) & \theta_{2}\left(t\right) & \theta_{3}\left(t\right) \end{array}\right]}^{T}\in R^{3\times 1},$$

τ is the vector of acting torques which can be described as

(22) $$\tau \left(t\right)={\left[\begin{array}{ccc} \tau_{1}\left(t\right) & \tau_{2}\left(t\right) & \tau_{3}\left(t\right) \end{array}\right]}^{T}\in R^{3\times 1},$$

$M\left(\theta (t)\right)\in R^{3\times 3}$ is the inertia matrix, $C\left(\theta \left(t\right),\dot{\theta }\left(t\right)\right)\in R^{3\times 3}$ is the Coriolis and centrifugal forces matrix, $g\left(\theta (t)\right)\in R^{3\times 1}$ represents the gravity force acting on the joints, θ(t), and $f\left(\dot{\theta }\left(t\right)\right)$ is the friction force on the joints, θ(t) [61,62,63,64].

**Figure 26:**
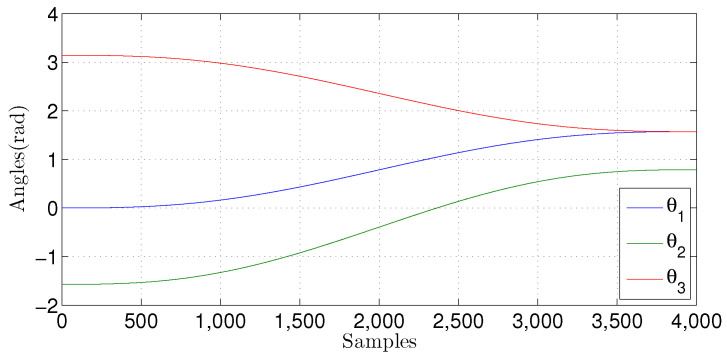
Trajectory of each rotatory joint of the Phantom Omni used to perform the simulations.

Figure 26 shows the angular position for each joint of the three-DOF Phantom Omni robotic manipulator, that is, $\theta_{1}$, $\theta_{2}$, and $\theta_{3}$. It is possible to observe the trajectory of each joint concerning its angular position as a function of the number of samples received.

The mean square error (MSE) between the actual and predicted data is used to define the reliability of the results generated by the proposal and can be defined as

(23) $$E_{qm}\left(X\right)=\frac{1}{N_{s}}\sum_{i=0}^{N_{s}-1}{\left(X\left(i\right)-(\hat{X})\left(i\right)\right)}^{2},$$

where $E_{q}m\left(X\right)$ is the value of the mean square error, $N_{s}$ is the number of samples, $(\hat{X})\left(i\right)$ is the *i*-th sample estimated value, and (X)(i) is the *i*-th sample current value.

The following subsections present the validation results for the implemented linear and nonlinear prediction techniques.

### 6.1. Linear Prediction Techniques

**Figure 27:**
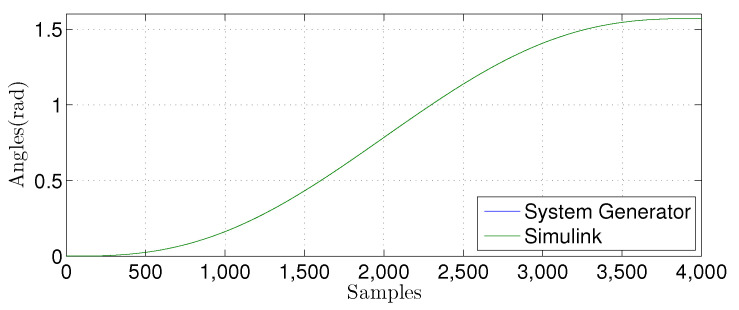
Comparison of the simulation results in Matlab Simulink and system generator for the linear regression technique with M=1.

**Figure 28:**
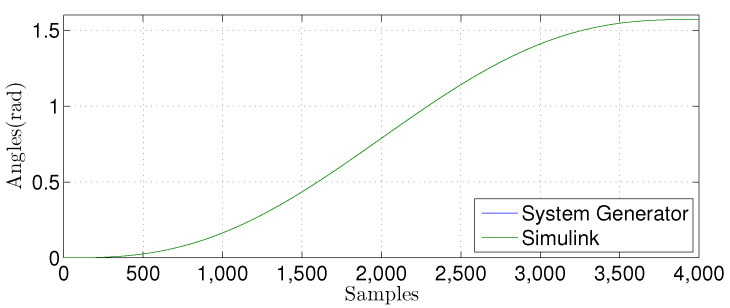
Comparison of the simulation results in Matlab Simulink and system generator for the linear regression technique with M=3.

**Figure 29:**
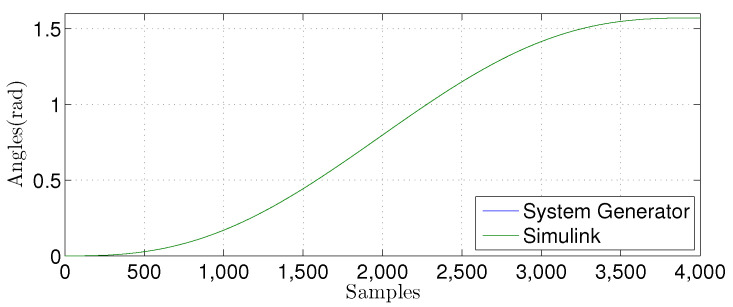
Comparison of the simulation results in Matlab Simulink and system generator for the linear regression technique with M=6.

**Figure 30:**
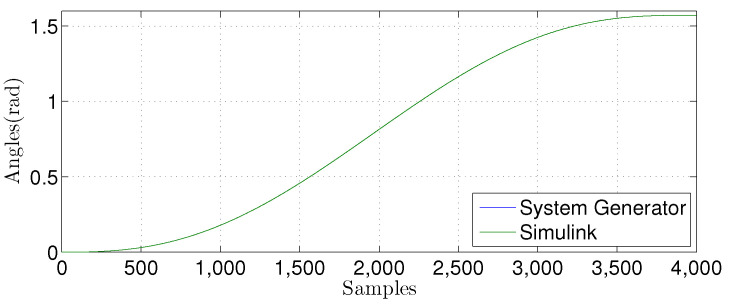
Comparison of the simulation results in Matlab Simulink and system generator for the linear regression technique with M=9.

We compared the $\theta_{1}\left(n\right)$ signal generated by our proposed FPGA architecture with one from a Matlab implementation for the linear prediction techniques. Figure 27, Figure 28, Figure 29 and Figure 30 show the results. We developed a Matlab version using a double-precision floating-point. In contrast, our hardware design uses a single-precision floating point. As can be observed, the results shown for the hardware implementation are similar to the Matlab version, despite reducing the hardware bit-width by half.

**Figure 31:**
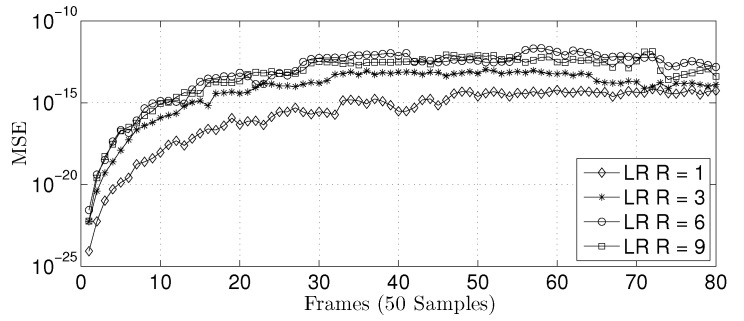
Comparison of the MSE value between the implemented linear prediction techniques.

Table 9 and Figure 31 present the MSE between the software (64-bit floating-point based on IEE754) and hardware (32-bit floating-point) implementations for the LR prediction techniques, using $N_{s}=4000$ data samples, 80 frames, and 50 samples per frame. As can be observed, the two implementations are equivalent, i.e., the MSE is significantly small.

### 6.2. Nonlinear Prediction Techniques

**Figure 32:**
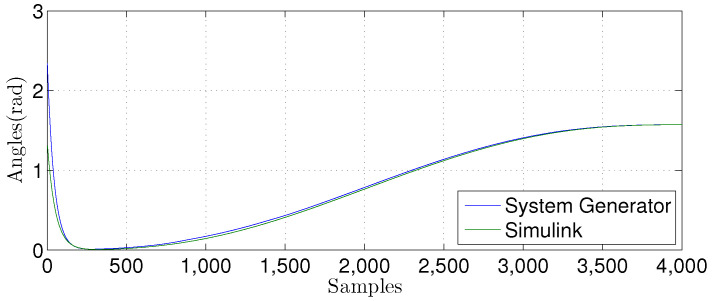
Comparisonof the simulation results in Matlab Simulink and system generator for the MLP-BP using W=14.

**Figure 33:**
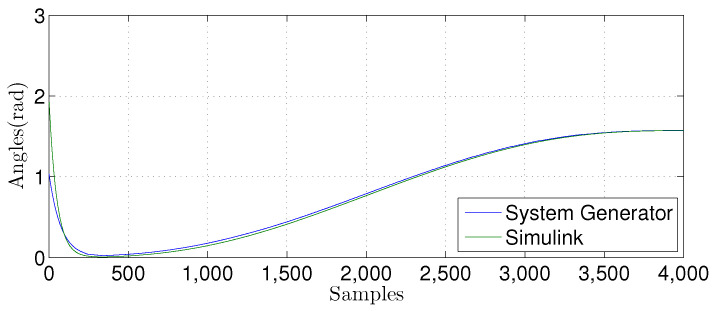
Comparison of the simulation results in Matlab Simulink and system generator for the MLP-BP using W=12.

**Figure 34:**
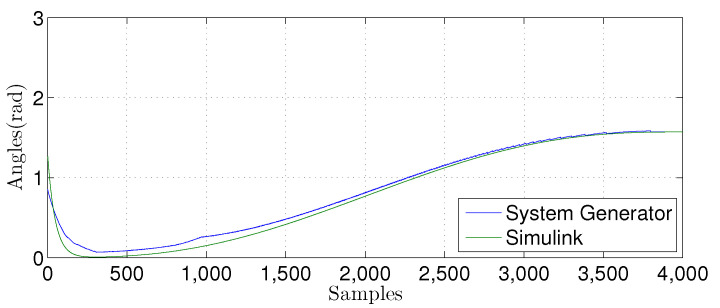
Comparison of the simulation results in Matlab Simulink and system generator for the MLP-BP with W=10.

For the nonlinear MLP-BP technique, we also compared the $\theta_{1}\left(n\right)$ signal. The results are presented in Figure 32, Figure 33 and Figure 34. We implemented a Matlab Simulink using a double-precision floating-point. The hardware uses fixed-point with the number of bits in the fractional part varying from W={10,12,14}. FPGA and Matlab implementations have similar behavior, showing that they are equivalent.

**Figure 35:**
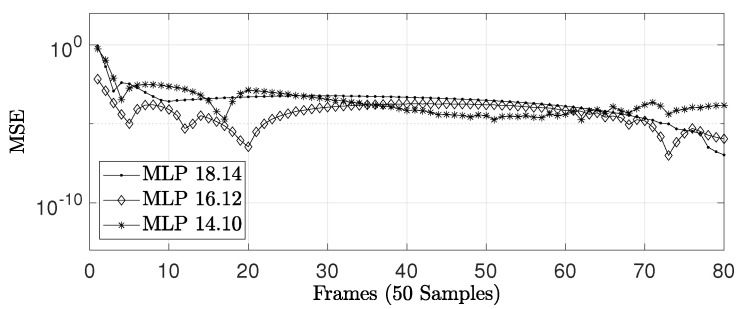
Comparison of the MSE value between MLP-BP implementations.

Afterwards, we performed an MSE analysis by varying the hardware bit-width from 18.14 to 16.12 and 14.10. The analysis was carried out for $N_{s}=4000$ data samples, 80 frames, and 50 samples per frame. Figure 35 and Table 10 show the resultant MSE. As can be observed, similarly to linear prediction techniques, the MSE between the software and hardware versions is also small for nonlinear techniques.

The proposed hardware implementations for prediction techniques have a similar response to the double-precision (64-bit) software implementation, even using fixed-point with fewer bits, such as 14.10. Furthermore, fewer bits may allow the implementation of the proposed method on hardware with limited capacity resources. Thus, the number of resources available could define the number of bits used to implement a technique.

After analyzing the MSE, it is possible to see that both linear and nonlinear techniques perform well in the current test scenario. However, as previously mentioned, linear-regression-based techniques may not be the most suitable for the TI landscape due to scalability issues seen in Section 5.1. Hence, in the following section, this work will focus on the results of the MLP-BP.

## 7. Comparison with State-of-the-Art Works

In this section, a comparison with state-of-the-art works is carried out for the following hardware key metrics: throughput, area occupation, and energy consumption. The implementations presented were developed on the Virtex-6 FPGA with T.W=14.10 bits.

### 7.1. Throughput Comparison

Table 11 shows the MLP processing speed and throughput for our work and other works in the literature. As can be seen, the columns present the number of implementations (NI), the fixed-point data precision (T.W), the MLP and MLP-BP processing speed, and the throughput in Msps.

The work proposed in [50] is an MLP with a 12–12–2 topology (twelve inputs, twelve neurons in the hidden layer, and two neurons in the output layer) deployed with a 24-bits fixed-point format. The MLP training is offline, and it reaches a throughput of 113.135Msps and 115.875Msps for the Virtex 6 XC6VLX240T and the Artix-7 XC7A100T FPGAs, respectively. The high performance achieved is due to the pipeline used in their proposed hardware design, reducing the system’s critical path and increasing the maximum frequency. Unlike [50], our proposal uses online training, and using a pipeline-based architecture is not feasible due to the chain of delays intrinsic to this approach that can reduce the sample’s accuracy during online training. Nevertheless, the throughput value of our architecture can improve as the number of implementations grows, increasing the number of samples processed per second without impacting its maximum clock.

The design proposed in [51] implements a 7–6–5 MLP with offline training on the Artix-7 35T FPGA. It achieved a throughput of 3.7Msps, but the number of clock cycles required to obtain a valid output reduces the throughput compared to other works. Meanwhile, the work presented in [52] proposes a 12–3–1 MLP on a Zynq-7000, also with offline training, capable of reaching a maximum throughput of 1.85Msps. The small throughput (compared to other works) may be related to the use of high-level synthesis (HLS), which usually results in a non-optimized implementation. The architecture presented in [53] is a 12–7–3 MLP with a 24-bit fixed-point data format and offline training. The maximum throughput achieved was 27.89Msps and 25.24Msps for the Virtex 6 XC6VLX240T and Artix-7 XC7A100T FPGAs implementations, respectively.

Table 12 presents a speedup analysis performed for all works presented in Table 11. The first column presents the NI in our architecture, while the second to seventh columns are the literature works compared with ours. $\frac{Throughput^{work}}{Throughput^{ref}}$ defines the speedup, where $Throughput^{work}$ represents the throughput of our proposal and $Throughput^{ref}$ represents the literature reference throughput. The results were obtained only for the MLP-BP implementation.

As shown in Table 12, the implementation seen proposed by [50] achieves a higher speedup. However, our proposal offers good scalability that allows increasing the NI and enables higher throughput, reducing this difference even with an implementation that uses online training embedded in the platform. Moreover, our approach reached a higher throughput than the other works, reaching speedup rates of up to 52×.

In addition, it is vital to mention that a higher frequency speed in MHz does not mean a higher throughput. Conversely, the throughput is commonly related to the parallelism degree. For example, the MLP speed in [51,52] have the lowest throughput even for a high-frequency speed (Table 11). In these cases, the speedup was up to 26× and 52× for [51] and [52], respectively.

In [53], the throughput value is 27.89 and 25.24Msps, for an MLP with offline training and NI = 1. Meanwhile, even implementing the training algorithm in hardware, our work achieves speedup rates of up to 3×.

In [50], a pipeline scheme reduces the system’s critical path and increases the throughput. However, it does not provide online training, which could reduce its performance. Meantime, our proposed architecture provides online training, adapting to different scenarios. In addition, it would not be feasible to use a pipelined scheme since the samples have a temporal dependence.

### 7.2. Hardware Area Occupation

The area occupation comparison was based on a hardware occupation ratio defined as

(24) $$R_{\mathrm{occupation}}=\left\{\begin{array}{cc} \frac{N_{\mathrm{hardware}}^{\mathrm{work}}}{N_{\mathrm{hardware}}^{\mathrm{ref}}} & ,\;\mathrm{for}\;N_{\mathrm{hardware}}^{\mathrm{work}}>0\;\mathrm{and}\;N_{\mathrm{hardware}}^{\mathrm{ref}}>0\\ \; & \\ \frac{1}{N_{\mathrm{hardware}}^{\mathrm{ref}}} & ,\;\mathrm{for}\;N_{\mathrm{hardware}}^{\mathrm{work}}=0\;\mathrm{and}\;N_{\mathrm{hardware}}^{\mathrm{ref}}>0\\ \; & \\ N_{\mathrm{hardware}}^{\mathrm{work}} & ,\;\mathrm{for}\;N_{\mathrm{hardware}}^{\mathrm{work}}>0\;\mathrm{and}\;N_{\mathrm{hardware}}^{\mathrm{ref}}=0\\ \; & \\ 1 & ,\;\mathrm{for}\;N_{\mathrm{hardware}}^{\mathrm{work}}=0\;\mathrm{and}\;N_{\mathrm{hardware}}^{\mathrm{ref}}=0. \end{array}\right..$$

The superscripts work and ref represent the resource information regarding our work and the compared work, respectively. Meanwhile, $N_{\mathrm{hardware}}$ represents the primitives, such as the number of LUTS, registers, multipliers, or the number of block random access memory (BRAM).

Table 13 shows the area occupation for our work and works in the literature. The second and third columns present the NI and fixed-point data precision (T.W). From the third to sixth columns, we present the number of LUTs (NLUT), the number of registers (NR), the number of multipliers (NMULT), and the number of BRAMs (NBRAM).

In [50], a total of 19,567 LUTs, 21,861 registers, 168 multipliers, and 26 BRAMs were used in the Virtex 6 XC6VLX240T, while the Artix-7 XC7A100T occupied a total of 19,732 LUTs, 21,659 registers, 168 multipliers, and 26 BRAMs. The memory usage can be attributed to implementing the sigmoid activation function. Meanwhile, our work uses the ReLU function; thus, it does not use memories.

The work presented in [51] uses an Artix-7 35T FPGA for the implementation, occupying 3466 LUTs, 569 registers, and 81 multipliers. The proposal shown in [52] uses 4032 LUTs, 2863 registers, 28 multipliers, and 2 BRAMs. The architecture proposed in [53] was implemented in two FPGAs using the sigmoid activation function, occupying 21,322 LUTs, 13,546 registers, 219 multipliers, and 2 BRAMs for the Virtex 6 XC6VLX240T FPGA, and 21,658 LUTs, 13,330 registers, 219 multipliers, and 2 BRAMs for Artix-7 XC7A100T.

Table 14, Table 15, Table 16 and Table 17 present the hardware ratio, $R_{occupation}$, regarding our proposed architecture.

As shown in Table 14, Table 15, Table 16 and Table 17, our proposal uses online training and implements up to six replicas of the same technique in parallel. For most cases, it requires fewer resources, evidencing efficient use of hardware. For a scenario where NI is 1, except for the works presented in [51,52], which have low throughput (see Table 11), our proposal maintains a good advantage over the other proposals. For a scenario where NI is 6, the present work has a high consumption of hardware resources compared to the other works. However, this is a strategy adopted to increase the throughput of the proposal. Furthermore, unlike other proposals, our design does not occupy any BRAMs as we use the ReLU function, thus improving the design’s scalability for flexible implementation in different scenarios, such as using TI systems with more DOFs, such as six or nine DOF.

### 7.3. Dynamic Power Consumption

Dynamic power is the primary factor for a digital circuit’s energy consumption. It can be expressed as

(25) $$P_{d}\propto N_{g}\times F_{\mathrm{clk}}\times V_{DD}^{2},$$

where $N_{g}$ is the number of elements (or gates), $F_{\mathrm{clk}}$ is the maximum clock frequency, and $V_{DD}$ is the supply voltage. Given that the operating frequency of CMOS circuits is proportional to the voltage [65], the dynamic power can also be described as

(26) $$P_{d}\propto N_{g}\times F_{\mathrm{clk}}^{3}.$$

The number of elements, $N_{g}$, can be defined by the FPGA primitives used to deploy the architecture, i.e., $N_{g}=\mathrm{NLUT}+\mathrm{NR}+\mathrm{NMULT}$.

Table 18 and Table 19 present the operating frequency and dynamic power analysis results regarding $N_{g}$. Concerning the dynamic power, we present the reduction rate, $S_{d}$, achieved by our proposal according to the following:

(27) $$S_{d}=\frac{N_{g}^{\mathrm{ref}}\times {\left(F_{\mathrm{clk}}^{\mathrm{ref}}\right)}^{3}}{N_{g}^{\mathrm{work}}\times {\left(F_{\mathrm{clk}}^{\mathrm{work}}\right)}^{3}},$$

where the $N_{g}^{\mathrm{ref}}$ and $F_{\mathrm{clk}}^{\mathrm{ref}}$ are the number of elements and the maximum clock frequency of the work we are comparing. At the same time, $N_{g}^{\mathrm{work}}$ and $F_{\mathrm{clk}}^{\mathrm{work}}$ are the number of elements and the maximum clock frequency of our work. Unlike the works in the literature, our hardware proposal uses a fully parallel layout, requiring one single clock cycle per sample processing. Therefore, the maximum clock frequency is equivalent to the throughput, $F_{\mathrm{clk}}^{\mathrm{work}}\equiv R_{s}$.

We assume that all proposals operate at the maximum frequency that the platform can reach. Thus, for an NI = 1, our design reduced power consumption by more than 1200× compared to the one proposed by [50]. Overall, our proposal reduced the power consumption compared to other work in most case scenarios. Therefore, IoT projects that require low power consumption can use our method without affecting their performance.

For NI = 6, we can observe a similar power consumption compared to [53] due to their proposal’s small clock value and not providing online training.

Lowering the use of BRAMs to zero is a highlight of this work. This reduction is possible due to the implementation of the ReLU function. Unlike other proposals that make use of functions, such as sigmoid, this strategy provides an advantage in terms of scalability of the proposal, which can be scaled to various scenarios without compromising the use of BRAMs. The fully parallel computing strategy proposed in the present work does not spend clock time accessing the RAM block, and this can increase throughput and decrease power consumption.

## 8. Conclusions

This work introduced a method for implementing prediction techniques in parallel to reduce the latency of TI systems using FPGA, thus enabling local devices to be used in conjunction with haptic devices. The hardware-based method minimized the data processing time of linear and nonlinear prediction techniques, showing that reconfigurable computing is feasible for solving complex TI problems.

We presented all the implementation details and the synthesis results for different bit-width resolutions and three different numbers of implementations in parallel (one, three, and six). In addition, the proposal is validated with a three-DOF Phantom Omni robotic manipulator and evaluated regarding hardware area occupation, throughput, and dynamic power consumption. In addition, we also presented comparisons with state-of-the-art works.

Comparisons demonstrate that a fully parallel approach adopted for linear regression and nonlinear prediction techniques can achieve high processing speed. However, linear regression techniques have low scalability and may not be a good path for the TI area. Nonlinear prediction techniques achieve a throughput of up to ≈52× while also reducing power consumption by ≈1300×. Furthermore, despite the high degree of parallelism, the proposed approach offers good scalability, indicating that the present work can be used in TI systems, especially for the nonlinear prediction techniques. 

## Figures and Tables

**Table 1:** Parameters used for implementation MLP-BP and RMLP-BP technique.

Parameter	Value
Number of nodes in layers	4–4–1
Activation function	ReLU
Training Algorithm	Backpropagation
Training mode	Online mode
η	0.008
α	0.0

**Table 2:** Synthesis results regarding the hardware area occupation, processing time, and throughput for the linear regression (LR) prediction technique, NI=1, and varying M from 1 to 9.

Method	NR	PR	NLUT	PNLUT	NMULT	PNMULT	$t_{s}$ (ns)	$R_{s}$ (Msps)
LR (M=1)	198	0.07%	3440	2.28%	9	1.17%	40.25	24.84
LR (M=3)	380	0.13%	5574	3.70%	9	1.17%	64.50	15.50
LR (M=6)	649	0.22%	8870	5.89%	9	1.17%	104.81	9.54
LR (M=9)	942	0.31%	11,762	7.80%	9	1.17%	142.16	7.03

**Table 3:** Synthesis results regarding the hardware area occupation, processing time, and throughput for the linear regression (LR) prediction technique, NI=3, and varying M from 1 to 9.

Method	NR	PR	NLUT	PNLUT	NMULT	PNMULT	$t_{s}$ (ns)	$R_{s}$ (Msps)
LR (M=1)	529	0.18%	9923	6.58%	27	3.52%	43.53	68.91
LR (M=3)	1075	0.36%	16,328	10.83%	27	3.52%	66.07	45.42
LR (M=6)	1886	0.63%	26,159	17.36%	27	3.52%	118.64	25.29
LR (M=9)	2764	0.92%	34,979	23.21%	27	3.52%	139.12	21,57

**Table 4:** Synthesis results regarding the hardware area occupation, processing time, and throughput for the linear regression (LR) prediction technique, NI=6, and varying M from 1 to 9.

Method	NR	PR	NLUT	PNLUT	NMULT	PNMULT	$t_{s}$ (ns)	$R_{s}$ (Msps)
LR (M=1)	1027	0.34%	19,649	13.04%	54	7.03%	42.42	141.48
LR (M=3)	2119	0.70%	32,457	21.53%	54	7.03%	66.81	89.82
LR (M=6)	3740	1.24%	52,146	34.60%	54	7.03%	104.75	57.30
LR (M=9)	5497	1.82%	69,595	46.18%	54	7.03%	171.32	35.04

**Table 5:** Synthesis results regarding the hardware area occupation, processing time, and throughput for the MLP prediction technique, varying NI and T.W.

NI	T.W	NR	PR	NLUT	PNLUT	NMULT	PNMULT	$t_{s}$ (ns)	$R_{s}$ (Msps)
1	18.14	547	0.18%	8141	5.40%	54	7.03%	54.24	18.44
	16.12	514	0.17%	7307	4.85%	54	7.03%	55.12	18.14
	14.10	431	0.14%	6575	4.36%	54	7.03%	52.94	18.89
3	18.14	1695	0.56%	24,483	16.24%	162	21.09%	64.42	46.57
	16.12	1590	0.53%	21,889	14.52%	162	21.09%	60.24	49.80
	14.10	1335	0.44%	19,729	13.09%	162	21.09%	55.39	54.16
6	18.14	3390	1.12%	48,520	32.19%	324	42.19%	63.95	93.82
	16.12	3180	1.05%	43,390	28.79%	324	42.19%	64.03	93.71
	14.10	2670	0.89%	39,718	26.35%	324	42.19%	61.86	96.99

**Table 6:** Synthesis results regarding the hardware area occupation, processing time, and throughput for the RMLP prediction technique, varying NI and T.W.

NI	T.W	NR	PR	NLUT	PNLUT	NMULT	PNMULT	$t_{s}$ (ns)	$R_{s}$ (Msps)
1	18.14	565	0.19%	8141	5.40%	54	7.03%	57.01	17.54
	16.12	530	0.18%	7303	4.85%	54	7.03%	55.87	17.90
	14.10	445	0.15%	6577	4.36%	54	7.03%	54.74	18.27
3	18.14	1,749	0.58%	24,455	16.23%	162	21.09%	63.66	47.13
	16.12	1,738	0.58%	21,885	14.52%	162	21.09%	56.99	52.64
	14.10	1,377	0.46%	19,725	13.09%	162	21.09%	56.37	53.22
6	18.14	3,498	1.16%	48,910	32.45%	324	42.19%	75.44	79.53
	16.12	3,276	1.09%	43,786	29.05%	324	42.19%	63.95	93.82
	14.10	2754	0.91%	39,460	26.18%	324	42.19%	60.77	98.73

**Table 7:** Synthesis results regarding the hardware area occupation, processing time, and throughput for the MLP module implemented without the BP and varying NI and the number of bits.

NI	T.W	NR	PR	NLUT	PNLUT	NMULT	PNMULT	$t_{s}$ (ns)	$R_{s}$ (Msps)
1	18.14	0	0.00%	1166	0.77%	25	3.26%	28.69	34.86
	16.12	0	0.00%	1061	0.70%	25	3.26%	27.30	36.64
	14.10	0	0.00%	956	0.63%	25	3.26%	28.41	35.20
3	18.14	0	0.00%	3510	2.33%	75	9.77%	32.65	91.90
	16.12	0	0.00%	3195	2.12%	75	9.77%	32.20	93.18
	14.10	0	0.00%	2880	1.91%	75	9.77%	30.72	97.65
6	18.14	0	0.00%	7020	4.66%	150	19.53%	34.36	174.63
	16.12	0	0.00%	6390	4.24%	150	19.53%	33.84	177.30
	14.10	0	0.00%	5760	3.82%	150	19.53%	31.81	188.62

**Table 8:** Synthesis results regarding the hardware area occupation, processing time, and throughput for the BP module and varying the number of bits.

NI	T.W	NR	PR	NLUT	PNLUT	NMULT	PNMULT	$t_{s}$ (ns)	$R_{s}$ (Msps)
1	18.14	475	0.16%	6411	4.25%	29	3.78%	29.16	34.29
	16.12	425	0.14%	5651	3.75%	29	3.78%	25.83	38.71
	14.10	350	0.12%	5316	3.53%	29	3.78%	25.16	39.74

**Table 9:** Mean square error (MSE) between the software implementation and the proposed hardware implementation for LR technique.

Method	MSE
LR (M=1)	$3.52\times 10^{-12}$
LR (M=3)	$4.52\times 10^{-11}$
LR (M=6)	$7.22\times 10^{-10}$
LR (M=9)	$3.99\times 10^{-10}$

**Table 10:** Mean square error (MSE) between the software and the proposed hardware implementations for nonlinear methods.

W	MSE
14	$5.36\times 10^{-7}$
12	$4.97\times 10^{-6}$
10	$2.93\times 10^{-4}$

**Table 11:** Throughput comparison with other state-of-the-art works.

Ref.	NI	Data Precision (T.W)	MLP Speed (MHz)	MLP-BP Speed (MHz)	Throughput (Msps)
[50]	1	24	113.14	-	113.14
			115.88	-	115.88
[51]	1	16.15	100	-	3.70
[52]	1	24.20	100	-	1.85
[53]	1	24	27.89	-	27.89
			25.24	-	25.24
This Work	1	14.10	35.20	18.89	18.89
	3		32.55	18.05	54.15
	6		31.44	16.17	97.02

**Table 12:** Speedup comparison of the MLP-BP implementation with other works.

NI	[50] 1	[50] 2	[51]	[52]	[53] 1	[53] 2
1	0.17×	0.16×	5.11×	10.21×	0.68×	0.75×
3	0.48×	0.47×	14.64×	29.27×	1.94×	2.15×
6	0.86×	0.84×	26.22×	52.44×	3.48×	3.84×

**Table 13:** Hardware occupation comparison with other works.

Ref.	NI	Data Precision (T.W)	NLUT	NR	NMULT	NBRAM
[50] 1	1	24	19,567	21,861	168	26
[50] 2	1	24	19,732	21,659	168	26
[51]	1	16.15	3466	569	81	0
[52]	1	24.20	4032	2863	28	2
[53] 1	1	24	21,322	13,546	219	2
[53] 2	1	24	21,658	13,330	219	2
This Work	1	14.10	6575	431	54	0
	3		19,729	1335	162	0
	6		39,718	2670	324	0

**Table 14:** Analysis of the ratio occupation for NLUT.

	NI	[50] 1	[50] 2	[51]	[52]	[53] 1	[53] 2
This Work	1	0.34×	0.33×	1.90×	1.63×	0.31×	0.30×
	3	1.01×	1.00×	5.69×	4.89×	0.93×	0.91×
	6	2.03×	2.01×	11.46×	9.85×	1.86×	1.83×

**Table 15:** Analysis of the ratio occupation for NR.

	NI	[50] 1	[50] 2	[51]	[52]	[53] 1	[53] 2
This Work	1	0.02×	0.02×	0.76×	0.15×	0.03×	0.03×
	3	0.06×	0.06×	2.35×	0.47×	0.10×	0.10×
	6	0.12×	0.12×	4.69×	0.93×	0.20×	0.20×

**Table 16:** Analysis of the ratio occupation for NMULT.

	NI	[50] 1	[50] 2	[51]	[52]	[53] 1	[53] 2
This Work	1	0.32×	0.32×	0.67×	1.93×	0.25×	0.25×
	3	0.96×	0.96×	2.00×	5.79×	0.74×	0.74×
	6	1.93×	1.93×	4.00×	11.57×	1.48×	1.48×

**Table 17:** Analysis of the ratio occupation for NBRAM.

	NI	[50] 1	[50] 2	[51]	[52]	[53] 1	[53] 2
This Work	1	0.04×	0.04×	1.00×	0.50×	0.50×	0.50×
	3	0.04×	0.04×	1.00×	0.50×	0.50×	0.50×
	6	0.04×	0.04×	1.00×	0.50×	0.50×	0.50×

**Table 18:** Analysis of the frequency regarding Ng.

Ref.	NI	Data Precision (T.W)	$F_{\mathrm{clk}}$	$N_{g}$
[50] 1	1	24	113.14	41,596
[50] 2	1	24	115.88	41,559
[51]	1	16.15	100.00	4116
[52]	1	24.20	100.00	6923
[53] 1	1	24	27.89	35,087
[53] 2	1	24	25.24	35,207
This Work	1	14.10	18.89	7060
	3		18.05	21,226
	6		16.17	42,712

**Table 19:** Analysis of dynamic power.

	NI	[50] 1	[50] 2	[51]	[52]	[53] 1	[53] 2
This Work	1	1265.90×	1358.91×	86.49×	145.48×	16.00×	11.90×
	3	482.61×	518.07×	32.97×	55.46×	6.10×	4.54×
	6	333.60×	358.11×	22.79×	38.34×	4.22×	3.13×

## Data Availability

Data available in https://github.com/natryus/Predicton-on-FPGA-for-TI (accessed on 29 March 2022).
